# Using the electrodermal activity signal and machine learning for diagnosing sleep

**DOI:** 10.3389/frsle.2023.1127697

**Published:** 2023-03-02

**Authors:** Jacopo Piccini, Elias August, María Óskarsdóttir, Erna Sif Arnardóttir

**Affiliations:** ^1^Reykjavik University Sleep Institute, School of Technology, Reykjavik University, Reykjavik, Iceland; ^2^Department of Engineering, Reykjavik University, Reykjavik, Iceland; ^3^Department of Computer Science, Reykjavik University, Reykjavik, Iceland; ^4^Landspitali–The National University Hospital of Iceland, Reykjavik, Iceland

**Keywords:** sleep, electrodermal activity, sleep stages, obstructive sleep apnea, machine learning

## Abstract

**Introduction:**

The use of the electrodermal activity (EDA) signal for health diagnostics is becoming increasingly popular. The increase is due to advances in computational methods such as machine learning (ML) and the availability of wearable devices capable of better measuring EDA signals. One field where work on EDA has significantly increased is sleep research, as changes in EDA are related to different aspects of sleep and sleep health such as sleep stages and sleep-disordered breathing; for example, obstructive sleep apnoea (OSA).

**Methods:**

In this work, we used supervised machine learning, particularly the extreme gradient boosting (XGBoost) algorithm, to develop models for detecting sleep stages and OSA. We considered clinical knowledge of EDA during particular sleep stages and OSA occurrences, complementing a standard statistical feature set with EDA-specific variables.

**Results:**

We obtained an average macro F1-score of 57.5% and 66.6%, depending on whether we considered five or four sleep stages, respectively. When detecting OSA, regardless of the severity, the model reached an accuracy of 83.7% or 78.4%, depending on the measure used to classify the participant's sleep health status.

**Conclusion:**

The research work presented here provides further evidence that, in the future, most sleep health diagnostics might well do without complete polysomnography (PSG) studies, as wearables can detect well the EDA signal.

## 1. Introduction

Electrodermal activity (EDA) is one of the longest-known and most accessible physiological signals (Boucsein, 2012). Electrodermal activity reflects changes in skin potential due to sweating, which, during sleep, has a thermoregulatory function. Eccrine sweat glands, the sweat glands that are activated during sleep (Boucsein, 2012), are innervated by the sympathetic nervous system (SNS) only, with no parasympathetic input (Baker, 2019). Despite this direct connection between EDA and the SNS during the night, the signal has been so far mostly used in studies of diurnal phenomena. For instance, it has been used for detecting stress (Zontone et al., 2019), epileptic seizures (Poh et al., 2010), and students' emotional engagement in classrooms (Di Lascio et al., 2018).

One of the main reasons for neglecting EDA in sleep studies is the complexity of the recorded signals. Long-term EDA recordings are susceptible to noise from various sources that cause artifacts in the signals, that is, sudden out-of-scale spikes; the most prominent sources of noise are body movements and poor skin-to-electrode connection. While in laboratory-controlled settings it is possible to log the patient's movements and to discard those signal segments when analyzing data, in free-living conditions, it is more difficult to do so. Because removing artifacts is important, much of the research on EDA signals has focused on automating their detection. Various methods have been proposed, often using supervised or unsupervised machine learning (ML) algorithms (Taylor et al., 2015; Hossain et al., 2022; Subramanian et al., 2022). Electrodermal activity has been only scarcely and only recently used for sleep staging or to infer sleep quality (Anusha et al., 2022; Gashi et al., 2022).

Abnormal sweating patterns may indicate the presence of various sleep disorders (Broman and Hetta, 1994; Idiaquez et al., 2022). In this work, we focused on sleep-breathing disorders, particularly obstructive sleep apnoea (OSA) (Jordan et al., 2014). Obstructive sleep apnoea causes unexpected SNS activity, resulting in frequent nocturnal sweating (Arnardottir et al., 2013). Despite the relationships between EDA and OSA has been studied (Lajos, 2004; Arnardottir et al., 2010), there is still a need for a quantitative model relating EDA and OSA.

In this paper, we applied supervised ML to EDA data to predict sleep stages and the presence of OSA. Currently, diagnosing it requires performing a full polysomnography (PSG) study in a laboratory setting, followed by manual scoring of the recordings. This procedure is time-consuming and can lead to atypical sleep patterns because of the differences between sleeping in a controlled environment, such as a sleep lab, and sleeping at home (Arnardóttir et al., 2021). We present an ML-based approach that uses features extracted from the EDA signal, recorded in a home-setting, to automatically detect sleep stages and OSA.

## 2. Materials and methods

We used a set of 60 full-night PSG recordings from participants in the Sleep Revolution Project (Arnardottir et al., 2022). We describe the cohort in detail in Table 1. The consent of the National Bioethics Committee and the Data Protection Authority of Iceland was granted for this study (VSN-21-070). All participants received and signed an informed consent for study participation.

**Table 1:** Dataset content according to the apnoea-hypopnoea index (AHI) or the oxygen desaturation index (ODI).

	**Non-OSA**	**Mild OSA**	**Moderate to severe OSA**
Number of participants (AHI)	19	24	17
Female participants (AHI)	47.4%	67.0%	29.4%
AHI	2.8 ± 1.3	10.0 ± 2.8	24.9 ± 10.5
BMI	25.8 ± 3.6	26.0 ± 3.6	25.8 ± 3.8
Age	36.2 ± 10.4	49.6 ± 14.7	52.0 ± 14.4
Percentage of epochs (AHI)	32.1%	39.0%	28.9%
Number of participants (ODI)	21	26	13
Female participants (ODI)	42.9%	61.5%	38.5%
ODI	1.5 ± 2.5	9.0 ± 2.5	24.1 ± 7.7
BMI	25.8 ± 3.8	27.7 ± 4.5	29.2 ± 2.8
Age	38.4 ± 12.4	48.2 ± 14.8	53.9 ± 14.0
Percentage of epochs (ODI)	33.9%	44.7%	21.4%

### 2.1. Instrumentation

Polysomnography (PSG) studies were recorded using A1 devices from Nox Medical (Reykjavik, Iceland). As the traditional PSG setup does not include EDA recordings, we added a channel for the EDA signal. A1 devices measured EDA at a sampling frequency of 200 Hz. For the measurement of the EDA signal, we used the same technique as in Arnardottir et al. (2010).

### 2.2. Sleep stage labeling

Sleep experts manually scored the electroencephalogram (EEG) and determined the sleep stage: wake (W), rapid eye movement (REM) sleep, sleep stage 1 (N1), sleep stage 2 (N2), and sleep stage 3 (N3). The scoring procedure was performed according to the American Academy of Sleep Medicine guidelines (Berry et al., 2020), using the Noxturnal software (Nox Medical, Reykjavik, Iceland). In this work, for detection, we considered both the above mentioned five stages or only four stages, by merging the N1 and N2 stages and relabeling them as light sleep. Additionally, we relabeled the N3 stage as deep sleep. The stages that we considered are then W, light sleep, deep sleep, and REM sleep, as is often done in the literature (Genzel et al., 2014). We report the distribution of sleep stages in Table 2.

**Table 2:** Distribution of sleep stages for 4 and 5 stages architectures.

**Wake**	**N1**	**N2**	**N3**	**REM**
12.2% ± 0.1	16.5% ± 0.1	32.5% ± 0.1	18.2% ± 0.1	20.6% ± 0.1
Wake	Light		Deep	REM
12.2% ± 0.1	49.0% ± 0.1		18.2% ± 0.1	20.6% ± 0.1

We report mean values and standard deviations.

### 2.3. Obstructive sleep apnoea labeling

Currently, OSA detection requires either manual scoring of a full PSG study or a home sleep apnoea testing, and the evaluation of two parameters: the apnoea–hypopnoea index (AHI) and the oxygen desaturation index (ODI) per hour of sleep (Berry et al., 2020). A shortcoming of the AHI is that it does not quantify one of the main consequences of OSA, which is oxygen desaturation. For this reason, sleep experts have defined the ODI value as the number of oxygen desaturation events ≥3% or ≥4% divided by the total sleep time (Chung et al., 2012; Berry et al., 2020). In this work, the sleep experts used 3% as threshold value.

We obtained a participant's OSA status from the manual scoring of PSG. We merged the moderate and severe OSA conditions to obtain three classes. To define them, we used the following modified version of the standard guidelines (AASM, 1999):

- Non-OSA: AHI <5,
- Mild OSA: 5 ≤ AHI <15,
- Moderate to severe OSA: AHI≥15.

We also classified the samples based on the ODI and computed the correlation between the two indexes and the EDA signal. Note that the ranges used for the ODI-based classification are the same as the ones for the AHI classification (Chung et al., 2012). Each epoch in an individual's data sample was labeled as either belonging to a non-OSA participant, one with mild OSA, or one with moderate to severe OSA. By epoch, we refer to a 30 s signal window. We adopted this time length to be consistent with the epochs' length used by sleep experts during manual scoring. Note that only seven samples were classified differently depending on whether we used the AHI or the ODI. Finally, we present the distribution of non-OSA, mild OSA, and moderate to severe OSA epochs in Table 1.

### 2.4. Signal pre-processing

From the Noxturnal software environment, we exported EDA signals using the EDF file format and imported them in MATLAB^®^ (MATLAB, 2022) for pre-processing and feature extraction. We down-sampled the original signal from 200 to 35 Hz to reduce the computational burden, following the guidelines presented in Braithwaite et al. (2013). We then pre-processed the original signal to obtain different kinds of data required by our detection algorithm.

First, because individual sweating patterns lead to significantly different-looking EDA signals (Boucsein, 2012), we computed the second-order polynomial best approximating the raw signal and subtracted it from the raw signal. Second, we applied a seventh-order Savitzky-Golay filter (Schafer, 2011) to the original signal to eliminate high-frequency contributions. We also applied a discrete wavelet transform (DWT) to the original signal. We computed the approximate and detailed discrete wavelet coefficients and soft thresholded the detail coefficients to remove possible recording noise (Coifman and Donoho, 1995). We then subtracted the Savitzky-Golay filtered signal from the discrete wavelet filtered signal; we referred to it as diffEDA.

Third, we computed the first and second-order derivatives of previously described three signals using a differentiator finite impulse response (FIR) filter. We used this method rather than a finite-differences scheme to prevent noise propagation. Particularly, we used a 50th-order filter with a passband frequency of 10 Hz and a stop-band frequency of 12.5 Hz. We disregarded the transient to avoid including artificial oscillations caused by applying the filter by discarding *N* = 50 samples. Note that, we denoted time derivatives by placing ∂_*t*_ or $\partial_{t}^{2}$ before the signal of interest; for example, we referred to the second time derivative of the de-trended signal as $\partial_{t}^{2}$detEDA.

**Figure 1:**
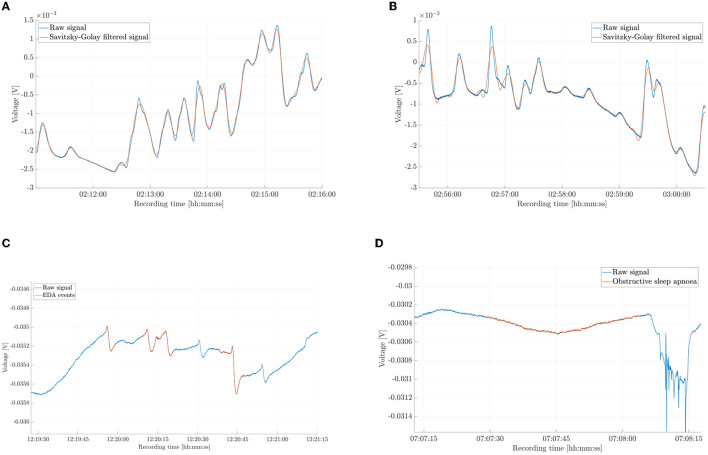
Different segments of the electrodermal activity (EDA) signal and of the Savitzky-Golay filtered signal from key phases of sleep. **(A)** Five minutes of raw and filtered signal during sleep stage 3 (N3). **(B)** Five minutes of raw and filtered signal during rapid eye movement sleep (REM). **(C)** EDA events (raw signal). **(D)** EDA during an obstructive sleep apnoea occurrence.

Figure 1 shows the complexity of the EDA signal. We show 5-min time windows of continuous N3 and REM sleep in Figures 1A, B, respectively. We then highlight EDA events in Figure 1C. Finally, we show the EDA signal during an OSA occurrence in Figure 1D.

### 2.5. Feature extraction and selection

We defined a feature set in the time-domain, frequency-domain, as well as time-frequency domain (these are wavelet-related variables) in a process called feature engineering (Verdonck et al., 2021). In addition to standard statistical features, we used number and energy content of EDA events and storms, as they are known to differ for different sleep stages (Sano et al., 2014) and OSA severity (Arnardottir et al., 2010). Electrodermal activity events are oscillations of the skin voltage of defined amplitudes and frequencies. We are particularly interested in the following three types of oscillations: positive/negative monophasic, biphasic, and triphasic. Electrodermal activity storms are time windows with high concentrations of events. The definition of storms has changed through time (Burch, 1965; Sano et al., 2014), we used an equivalent definition to the one given by Sano and colleagues, that is, a timespan of at least 1 min with a minimum of two EDA events. Particularly, we used the algorithm developed in Piccini et al. (2023) to detect EDA events and storms. Thereafter, we computed the normalized number of samples within either an EDA event or storm, together with their Euclidean norms. Additionally, we added sex as a categorical feature to complete the set of variables and normalized the features across individuals. The full feature set is shown in Table 3.

**Table 3:** Set of variables extracted from the electrodermal activity (EDA) signal.

**Index**	**Signal**	**Computed features**
1–18	EDA detEDA	Mode, median, maximum of absolute value, line length, 10^th^ quantile, 75^th^ quantile, singular value decomposition (SVD) entropy, non-linear energy, Shannon entropy
19–34	∂_*t*_EDA, $\partial_{t}^{2}$EDA ∂_*t*_detEDA, $\partial_{t}^{2}$detEDA	Mean value, variance, median value, numbers above zero
35–40	EDA detEDA	Maximum power spectral density (PSD) estimate, frequency of the maximum PSD estimate, Fisher's g (Posada-Quintero et al., 2016)
41–64	EDA detail coefficients decomposition levels (DL) 1—4	Maximum, mean, standard deviation, median, Euclidian norm, normalized numbers above zero
65–70	EDA detEDA	Lyapunov exponent, maximum value of the upper envelope, minimum value of the lower envelope
71–72	diffEDA	Sum of cross-correlation, maximum convolution value
73–76	EDA	Normalized number of event samples, normalized event energy, normalized number of storm samples, normalized storm energy
77	Individual	Sex

Finally, after training and testing the model on the complete variable set, we investigated whether we could reduce the feature set dimension by analyzing intra-variable correlation. We identified correlated features by computing the pairwise Pearson correlation coefficient *r*. We then reduced the dimension of the feature set by retaining only one of the correlated variables. We looked at the correlation matrix to identify the threshold value *r*_*th*_.

### 2.6. Training procedure

Sleep stages are not equally distributed during the night, this asymmetry caused a significant imbalance in our dataset and affected model performance. To reduce the negative impact of this effect, we performed synthetic minority oversampling (SMOTE) (Chawla et al., 2002), that is, we generated artificial samples for the minority classes to alleviate the bias toward the most dominant class. We then trained models using the extreme gradient boosting (XGBoost) algorithm (Chen and Guestrin, 2016), since a gradient boosting algorithm was recently used in a similar application with promising results (Gashi et al., 2022).

We applied different validation methods. We either used leave-one-subject-out (LOSO) validation (Hastie et al., 2009), where we alternately left out one sample and used the other 59 samples as training data, or we did as previously and in addition, we trained the model using randomly selected 25% of the epochs from the left-out subject's night (Personalized). We always used the same seed for reproducibility. After this random sampling, we applied the SMOTE algorithm to the training data. We evaluated the OSA model only by means of the LOSO scheme. We did so, because of the way that we labeled the data for OSA detection, see Section 2.3.

### 2.7. Evaluation metrics

We computed different measures to evaluate the models' performances. All indices were obtained using scikit-learn (Pedregosa et al., 2011). F1 and recall scores were used to evaluate the sleep staging performances. While F1-score is a commonly used measure in ML applications, we used the recall score to account for the significant class imbalance (Gashi et al., 2022). Recall score is the ratio between true positives and the sum of true positives and false negatives and, thus, a measure for the number of relevant objects detected by the algorithm. The F1-score is the harmonic mean of precision and recall scores and is used in classification problems with imbalanced datasets, as the precision score on its own may be misleading. As we dealt with a multi-class classification problem, we used the macro version of both parameters; the macro F1-score is the average of all F1-scores, and the macro recall is the average of all recalls. For the remainder of the paper, we referred to the macro F1-score and macro recall value simply as F1-score and recall.

For the OSA model, we used the F1-score and accuracy values. Accuracy is the ratio between the number of correctly identified epochs and the total amount of epochs. In addition to these two measures, we evaluated the three-class algorithm's ability to distinguish between non-OSA participants and those with OSA. To do this, we considered all OSA epochs as equivalent, which made the classification problem a binary one; we then computed the accuracy score and referred to it as the adjusted accuracy score. We did not include the recall for OSA models' evaluations, as it deals with all misclassifications in the same way. Particularly, misclassifications between severe and mild OSA conditions and between non-OSA and OSA conditions have different clinical meanings.

### 2.8. SHapley Additive exPlanations

To evaluate the contribution of each training variable, we used SHapley Additive exPlanations (SHAP) (Lundberg and Lee, 2017). The technique was developed in game theory and only recently adapted to ML interpretability applications (Lundberg et al., 2018). To find the SHAP value of the *i*-th variable, we computed the predictions for all possible feature combinations with and without the *i*-th variable. The SHAP value is then the average of the contributions of the *i*-th variable to each prediction (Molnar, 2022).

## 3. Results and discussion

### 3.1. Feature reduction

**Figure 2:**
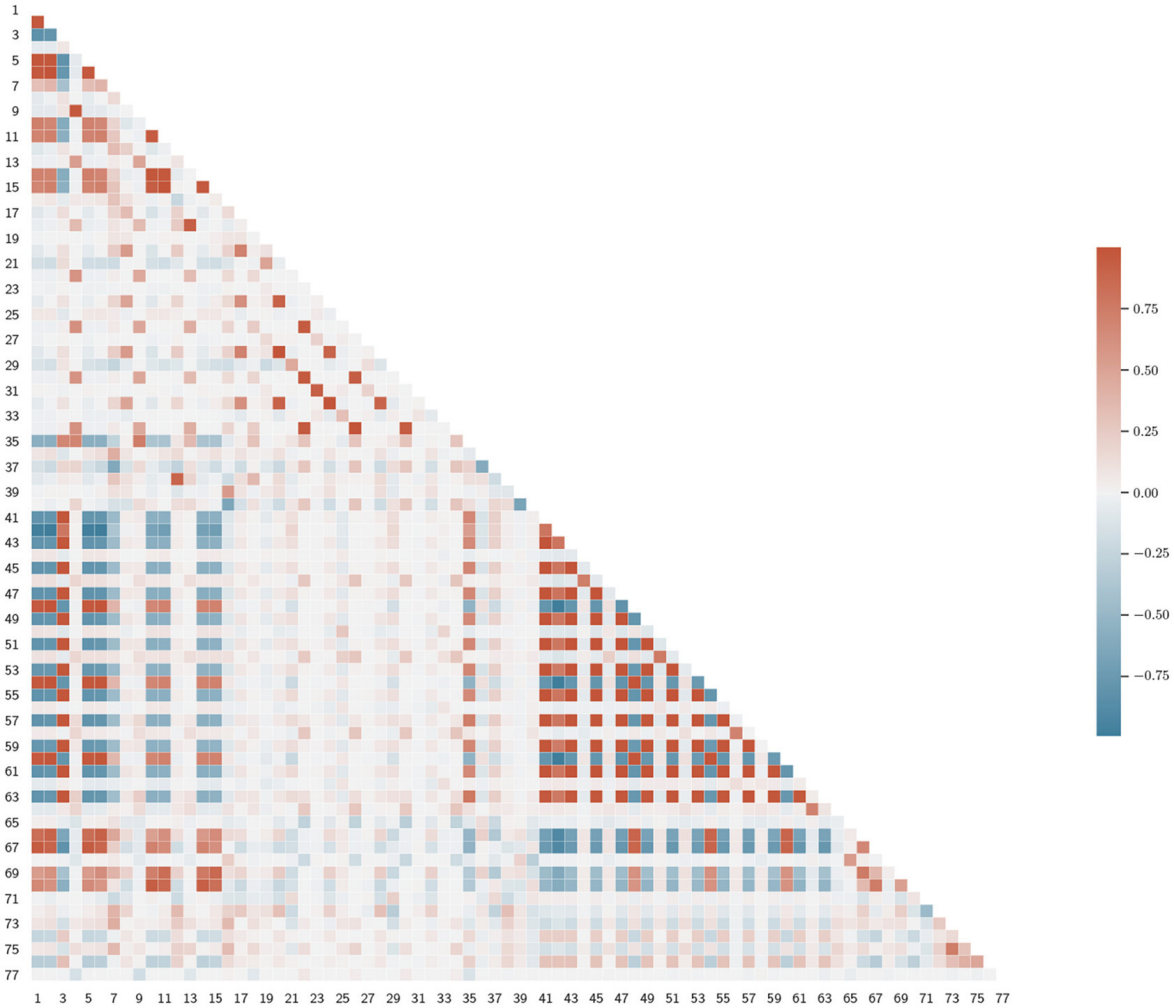
Representation of the lower triangular feature correlation matrix. We denoted the variables by index, as in Table 3.

Before presenting the models' performances, we offer an analysis of the feature set reduction process; for the sake of notation, we refer to variables by index, as in Table 3. We identified three main clusters of correlated variables by looking at the graphical representation of the correlation matrix (Figure 2). The first one involves features 1–15, which are statistical measures, in the time domain, of EDA and detEDA signals. The second one is a large cluster encompassing features 41–64, variables obtained in the time-frequency domain. Finally, non-linear features 65–70 also show meaningful correlation patterns.

**Figure 3:**
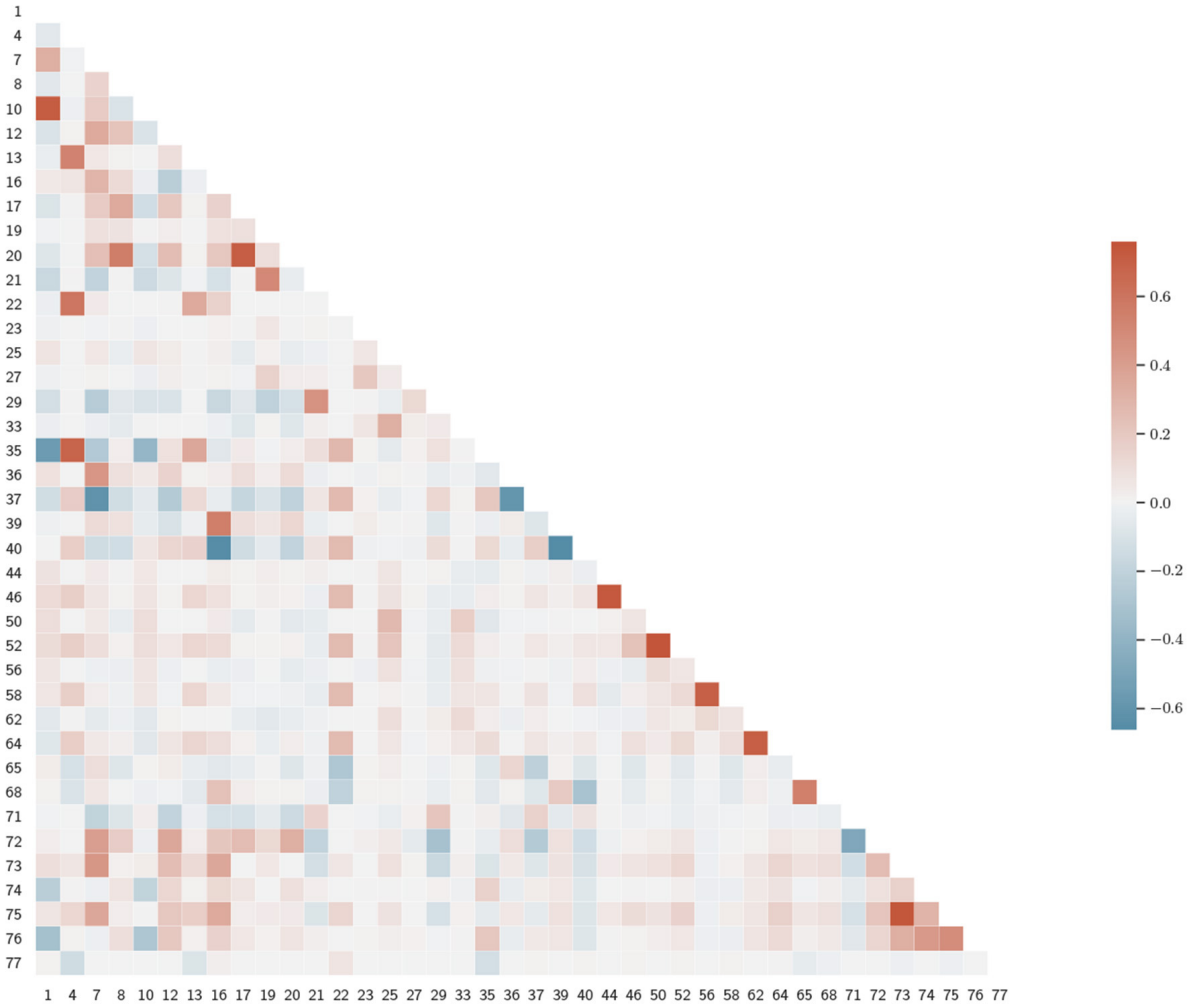
Correlation matrix for the reduced feature set. Variables are denoted by index, as in Table 3.

After setting *r*_*th*_ = 0.8, we reduced the number of correlated variables. We decided which feature to eliminate as follows: first, we computed the correlation coefficients between the *i*-th feature and the remaining ones, then we eliminated the *j*-th feature, if *r*_*i,j*_>*r*_*th*_, where *r*_*i,j*_ is the Pearson correlation coefficient between the *i*-th and the *j*-th variables and *j*>1. We started at *i* = 1. In this way, we obtained a reduced set of 40 features, which we present in Table 4. We opted not to decrease further the *r*_*th*_-value, as the resulting feature set did not present any significant clusters, see Figure 3. Also, lower values of *r*_*th*_ may result in worse classification performances.

**Table 4:** Optimized feature set for the sleep staging models, *r*_*th*_ = 0.8.

**Signal**	**Computed features**
EDA	Mode, maximum of absolute value, line length, singular value decomposition (SVD) entropy, non-linear energy, Lyapunov exponent, maximum power spectral density (PSD) estimate, frequency of the maximum PSD estimate, Fisher's g (Posada-Quintero et al., 2016)
detEDA	Mode, maximum of absolute value, line length, singular value decomposition (SVD) entropy, non-linear energy, Lyapunov exponent, requency of the maximum PSD estimate, Fisher's g (Posada-Quintero et al., 2016)
∂_*t*_EDA	Mean, variance, median, number above zero
$\partial_{t}^{2}$EDA	Mean, median
∂_*t*_detEDA	Mean, median
$\partial_{t}^{2}$detEDA	Median
EDA detail coefficients decomposition levels (DL) 1–4	Median, normalized numbers above zero
diffEDA	Sum of cross-correlation, maximum convolution value
EDA	Normalized number of event samples, normalized event energy, normalized number of storm samples, normalized storm energy
Individual	Sex

### 3.2. Interpretation of sleep staging

**Figure 4:**
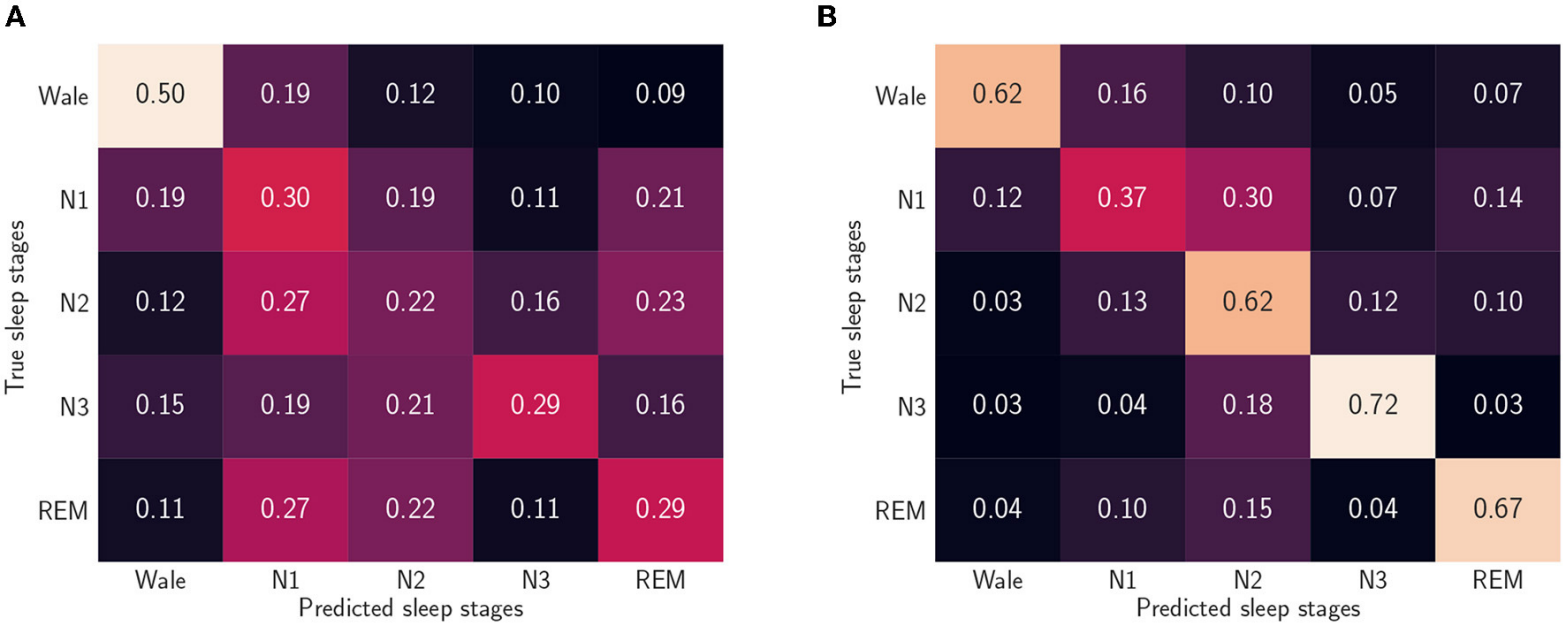
Normalized confusion matrices when we consider five sleep stages and use the reduced feature set. **(A)** Leave-one-subject-out (LOSO). We trained the algorithm without including data from the left out participant. **(B)** Personalized model. In addition to the 59 participants training set, we used randomly picked 25% epochs of the test participants.

**Figure 5:**
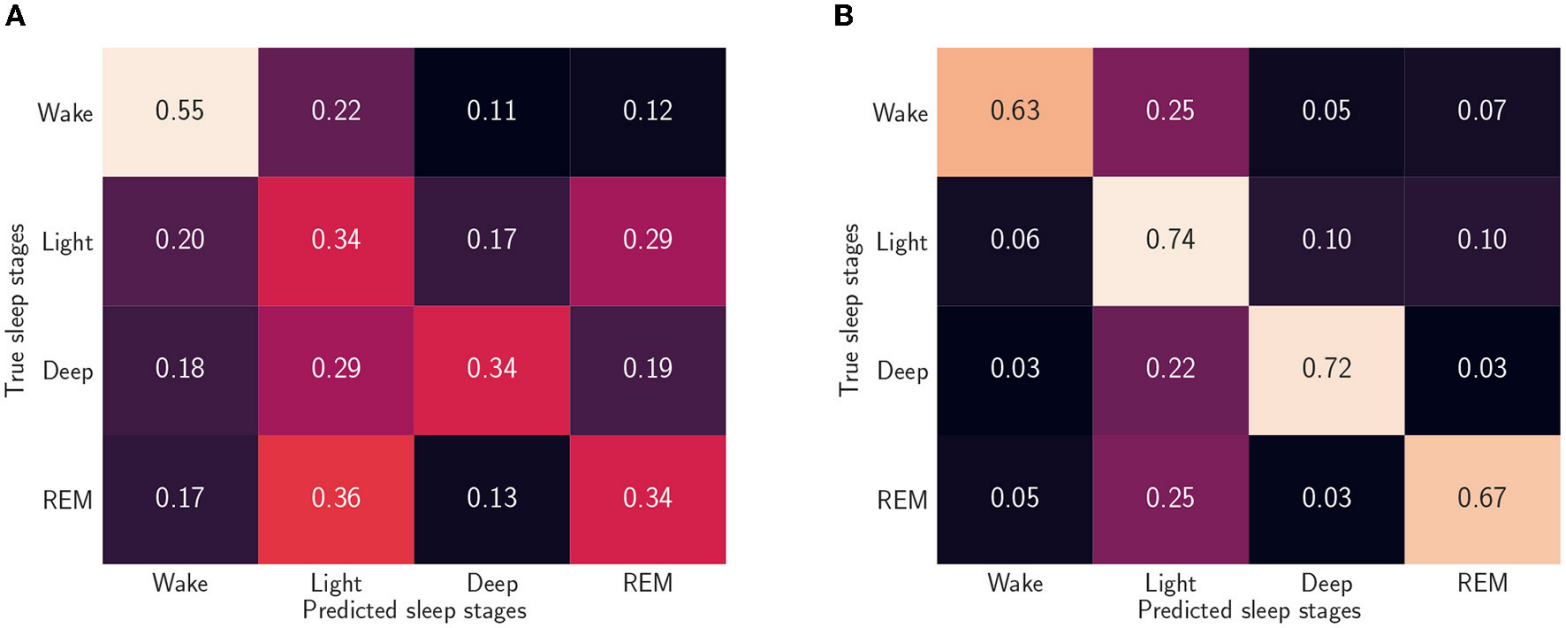
Normalized confusion matrices when we consider four sleep stages and use the reduced feature set **(A)** Leave-one-subject-out (LOSO). We trained the algorithm without including data from the left out participant. **(B)** Personalized model. In addition to the 59 participants training set, we used randomly picked 25% epochs of the test participants.

We summarized the models' performances in Table 5, where the F1-scores and recall values are reported. Our results suggest a need for personalized models (Óskarsdóttir et al., 2022). A possible explanation for the relatively poor performance is that different brain regions can be in different sleep stages at the same time. For instance, sweat glands' activation signals and, thus, EDA, are generated in the hypothalamus (Rothhaas and Chung, 2021), while the EEG, used to manually label sleep stages, measures neocortex activity, and it is known that the two brain areas can be in different sleep stages (Guthrie et al., 2022). However, personalizing the LOSO-based model with a small number of epochs from the left-out sample dramatically improves the algorithm, see also Figures 4, 5, which show confusion matrices normalized such that the sum of each row equals one.

**Table 5:** Summary of sleep staging performance for, both, four stages, and five stages classification.

**No. stages**	**r*th***	**Leave-one-subject-out**		**Personalized**	
		**Macro F1-score**	**Macro recall score**	**Macro F1-score**	**Macro recall score**
Five stages	0.8	27.3%	32.4%	57.5%	58.0%
Four stages	0.8	32.8%	39.7%	66.6%	66.9%

By looking at the confusion matrix in Figure 4B, we concluded that the personalized model cannot characterize the N1 sleep stage using only EDA. Furthermore, N1 detection appeared to be a cumbersome task even when other ML methods and other signals were used, such as EEGs, electrooculograms (EOGs), and electromyograms (EMGs) (Chambon et al., 2018; Korkalainen et al., 2019). A similar disagreement in determining the sleep stage was also found when comparing different manual scorings (Magalang et al., 2013). However, the detection of slow wave sleep (SWS) phases, that is, deep sleep and N3 stage, and REM sleep phases worked well for both models. This was expected, since these are the phases with the most distinct EDA patterns. Notably, by looking at Figure 4B, we can conclude that, based on EDA, the N3 stage is more similar to the N2 stage than any other sleep stage.

**Figure 6:**
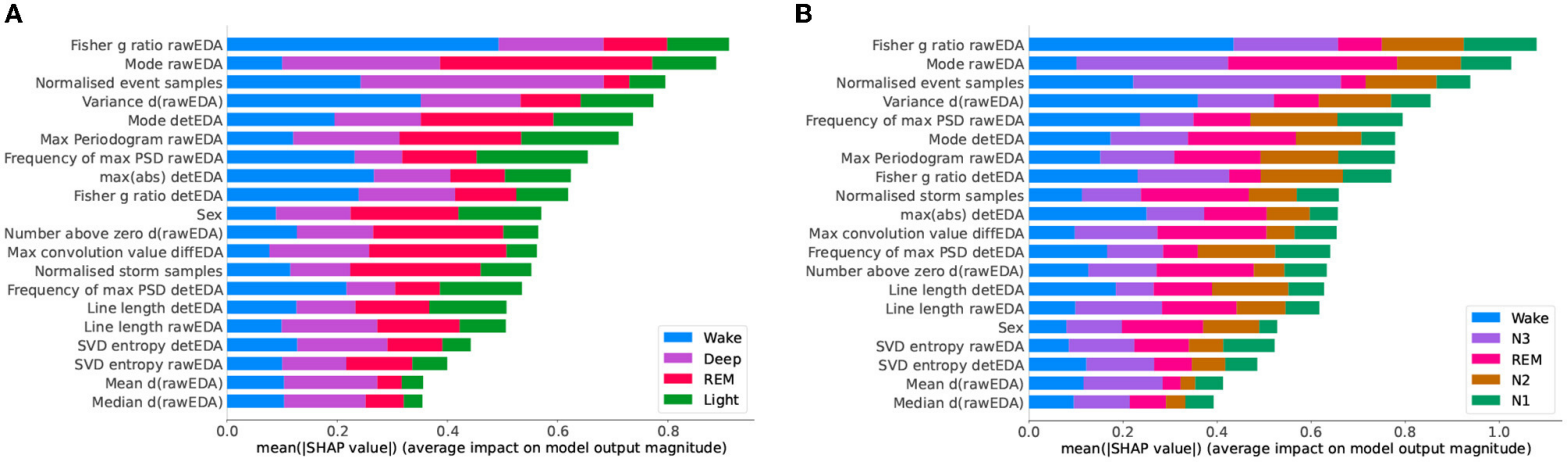
SHapley Additive exPlanations (SHAP) values of the sleep staging model trained using the leave-one-subject-out (LOSO) scheme and the reduced feature set. **(A)** Four sleep stages: wake (W), light sleep, deep sleep, rapid eye movement (REM) sleep. **(B)** Five sleep stages: wake (W), sleep stage 1 (N1), sleep stage 2 (N2), sleep stage 3 (N3), rapid eye movement (REM) sleep.

Finally, we offer a graphical interpretation of the sleep staging model, trained on the reduced dataset, through the SHAP values of the 20 most influential variables, see Figure 6. It is worth noting that both models considered the number of EDA events to be highly relevant for N3 stage, see Figure 6. The models also predict a significant relationship between EDA storms and REM sleep. Indeed, it is known that EDA activity increases in the third cycle of REM sleep (Boucsein, 2012).

### 3.3. The need for personalization in sleep staging

Several physiological considerations support the need for personalization in EDA-based sleep staging. Nocturnal sweat, the principal cause of changes in skin electrical properties, is secreted to lower the core body temperature (CBT) (Baker, 2019). However, the thermoregulation process depends on a large number of factors, for example, age, BMI, sex, skin hydration, eccrine sweat gland concentration, and environmental conditions (Speakman, 2018; Grosiak et al., 2020; Yanovich et al., 2020). All the factors mentioned significantly impact sweat and, consequently, the EDA signal. Furthermore, the latter is also affected by subject-dependent brain dynamics.

It is not straightforward to decide which personal subset of epochs to choose, as different EDA patterns arise in different parts of the night; for example, EDA events are more frequent in REM sleep during the last sleep cycle (Boucsein, 2012), while rarer in other REM sleep periods. Furthermore, differences in sleep cycle duration caused by age and OSA condition, among other factors, may hinder the beneficial effect of the algorithm's personalisation. Because of this, we opted for a fixed-seed random-pick approach.

### 3.4. Interpretation of the OSA model

To evaluate the models' ability to distinguish between non-OSA persons and those with either mild or moderate to severe OSA, we used average values of the accuracy score, the F1-score, and the adjusted accuracy score. We also evaluated a binary classification problem, where participants either had OSA or not, for which we refrained from calculating the adjusted accuracy score. We present the results as we did for the sleep staging models in Table 6. They show that OSA severity determined through the EDA signal rather follows the classification obtained by using the ODI rather than the AHI. A possible explanation for this behavior is how the ODI value divides the participants. Looking at Table 1, we observed that while both the indexes found the mean age to increase with the OSA severity, in ODI classification BMI values also increased with OSA severity. Lower BMI values have been associated with lower mean temperature values, (Waalen and Buxbaum, 2011), which may result in less need for thermoregulation. Consequently, simpler sweating patterns may be observed, which are better learned by the algorithm.

**Table 6:** Results for obstructive sleep apnoea (OSA) detection, based on the apnoea-hypopnoea index (AHI) or on the oxygen desaturation index (ODI).

**OSA structure**	**r_*th*_**	**Mean accuracy score**	**Macro F1-score**	**Adj. accuracy score**
AHI—non-OSA vs. OSA	0.8	75.7%	65.6%	–
ODI—non-OSA vs. OSA	0.8	82.0%	67.7%	–
AHI—Three groups	0.8	54.8%	32.9%	78.4%
ODI—Three groups	0.8	54.8%	32.9%	83.7%

**Figure 7:**
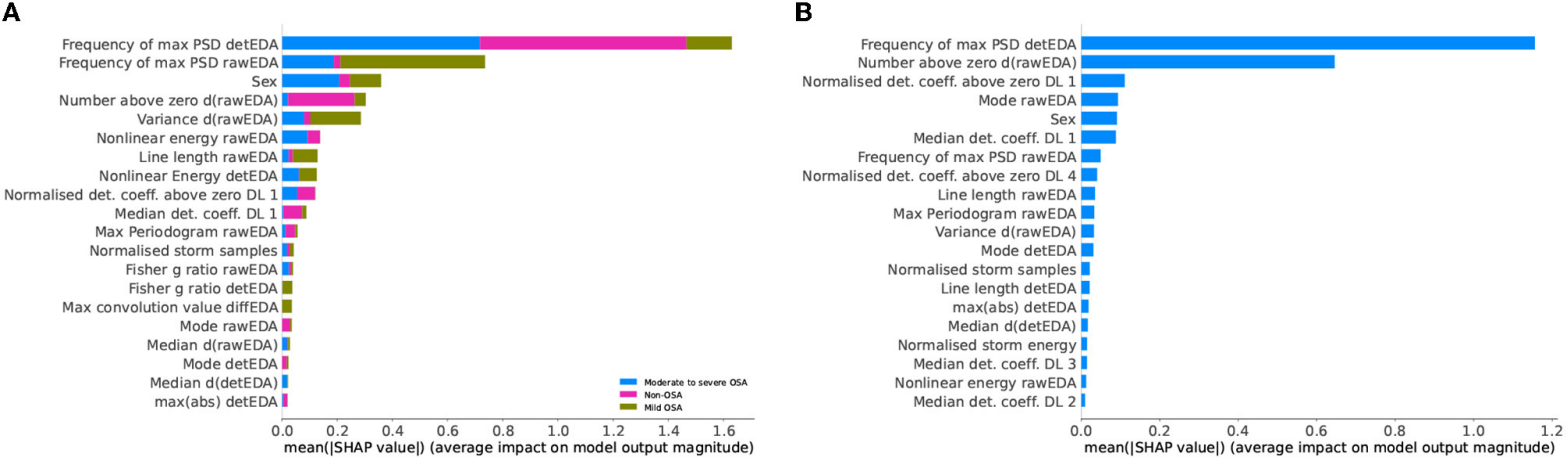
SHapley Additive exPlanations (SHAP) values of the obstructive sleep apnoea (OSA) detection model based on apnoea-hypopnea index (AHI) values and the reduced feature set. **(A)** Three-class classification problem: participants with no, mild, or moderate to severe OSA. **(B)** Binary classification problem: non-OSA participants and those with OSA.

**Figure 8:**
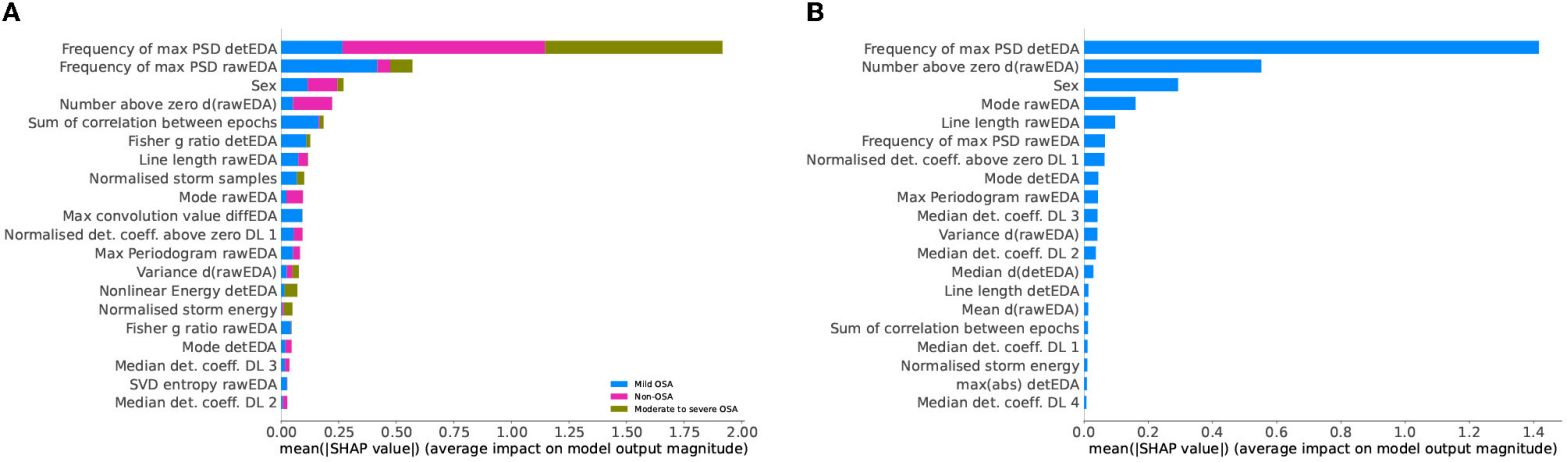
SHapley Additive exPlanations (SHAP) values of the obstructive sleep apnoea (OSA) detection model based on oxygen desaturation index (ODI) values and the reduced feature set. **(A)** Three-class classification problem: participants with no, mild, or moderate to severe OSA. **(B)** Binary classification problem: non-OSA participants and those with OSA.

In Figures 7, 8, we used the SHAP values to present the effect of each variables on the different classification problem. Both three-class models choose normalized storm samples as one of the most significant variables, which relates well to the literature (Arnardottir et al., 2010).

### 3.5. Feature selections comparison

Out of the 77 extracted variables, only eight appear in all models' top 20 most important features. They are EDA mode, ∂_*t*_EDA variance, detEDA mode, EDA maximum power spectral density (PSD) estimate, EDA frequency of the maximum PSD estimate, ∂_*t*_EDA normalized numbers above zero, detEDA frequency of the maximum PSD estimate, and biological sex. The seven numerical variables are computed from two signals, that is, raw and de-trended EDA and the derivative of the raw signal; this subset is composed of variables spanning multiple domains, particularly time, frequency, and EDA-specific. This variety confirms the need to consider different dynamical behaviors and EDA-related phenomena when using this signal. The most common specific feature is the number of EDA storm samples, which is amongst the top 20 most important features for all models, except for the two-class ODI-based OSA classification problem. However, in the latter problem, normalized storm energy is considered a relevant feature. Works trying to relate EDA and OSA are scarce and based mainly on subjective night sweats reports (Nigro et al., 2022). Although it is well-established that OSA symptomatology includes abnormal sweating episodes (Arnardottir et al., 2010, 2013), there needs to be more understanding of the relationship between OSA and EDA events and storms. Our work concludes that evaluating EDA storms, their lengths or energies, is more decisive in detecting OSA, particularly severe expressions, than evaluating EDA events. This conclusion holds for OSA classifications based on both AHI and ODI severity.

## 4. Conclusion and future work

The presented work aimed at detecting sleep stages and OSA severity using only the EDA signal. Recently, Anusha and colleagues presented an ML algorithm for identifying the sleep stage of the hypothalamus, the brain region directly responsible for thermoregulation during sleep (Anusha et al., 2022), while, Gashi and colleagues presented a similar algorithm based on EDA that is able to detect wake/sleep stages and high/low sleep quality (Gashi et al., 2022). Latter algorithms are based on self-reported annotations. Despite these significant results, more research on the relationship between EDA and neocortex activity is needed. Our work is the first one, in which neocortex sleep stages are predicted solely based on EDA. In the first part of this research work, we presented a sleep staging algorithm that is particularly accurate in detecting those sleep stages, where specific EDA patterns are known to occur, which are N3 and REM sleep. In the second part, we focused on OSA detection. By using the EDA signal, we distinguished non-OSA participants from those with OSA with reasonable accuracy.

Our work has three main limitations. The first one is that the raw signal was recorded at 200 Hz, an unattainable sampling frequency for current wearables. However, the signal was significantly downsampled, to 35 Hz, before it was handled. Since EDA events occur in the frequency band [0.25–3 Hz] for endosomatic recordings, like the ones used in this study, further downsampling might potentially be performed without a significant loss of information, which we leave for future work. The second limitation is that the sleep staging algorithm requires a certain amount of individual data manually scored by a sleep expert. While this prevents the sleep staging model from being user-independent and, thus, might limit its use in wearables, in clinical studies, requiring only a small part of the signal to be manually scored significantly saves time and cost. Moreover, our work adds to the body of evidence on how crucial it is to include knowledge about sleep processes in ML models. A final limitation is the participants' significant ranges in age and BMI within a relatively small sample size. While the participants' diversity ensured to obtain general models, it also prevented the algorithm from learning patterns specific to a particular group, for example, individuals of the same biological sex and of similar age. Future studies may overcome this last limitation by using a more selected cohort or by considering the body temperature signal, therefore addressing the differences in mean body temperature due to various aspects such as age, sex, and BMI.

To improve on the reported results, in the future, we will also include additional signals obtainable through wearables, such as acceleration and skin temperature. Doing so might reduce the need for individual tuning of the algorithm and allow it to identify other sleep stages more accurately. More precise sleep staging based on data obtained from wearables will allow the estimation of more advanced sleep parameters used in sleep diagnostics, such as total sleep time and sleep efficiency. Finally, since the algorithm labels each epoch as “non-OSA” or “OSA-prone,” it will be possible to track a potential onset or worsening of sleep-disordered breathing. By adequately characterizing the development of OSA symptoms, it will be possible to define a threshold that will lead to suggesting to seek professional advice when exceeded.

## Data availability statement

The datasets used for this study is not publicly available due to General Data Protection Regulation (GDPR) reasons. Requests to access the datasets should be directed to jacopop@ru.is.

## Ethics statement

The study received the approval of the National Bioethics Committee and the Data Protection Authority of Iceland (Sleep Revolution VSN-21-070). The patients/participants provided their written informed consent to participate in this study.

## Author contributions

JP: conceptualization, methodology, software, visualization, and writing. EA and MÓ: conceptualization, methodology, supervision, reviewing, and editing. ESA: supervision, reviewing, editing, and funding acquisition. All authors contributed to the article and approved the submitted version.
